# Glucose-Dependent Regulation of *NR2F2* Promoter and Influence of SNP-rs3743462 on Whole Body Insulin Sensitivity

**DOI:** 10.1371/journal.pone.0035810

**Published:** 2012-05-14

**Authors:** Marie Boutant, Oscar Henrique Pereira Ramos, Cécile Lecoeur, Emmanuel Vaillant, Julien Philippe, Pili Zhang, Anaïs Perilhou, Beatriz Valcarcel, Sylvain Sebert, Mario-Ritta Jarvelin, Beverley Balkau, Donald Scott, Philippe Froguel, Martine Vaxillaire, Mireille Vasseur-Cognet

**Affiliations:** 1 Department of Endocrinology, Metabolism and Cancer, Cochin Institute, CNRS (UMR 8104), Paris, France; 2 INSERM U1016, Paris, France; 3 Université Paris Descartes, Sorbonne Paris Cité, Paris, France; 4 Centre National de la Recherche Scientifique (CNRS)-Unité mixte de recherche (UMR) 8199, Lille Pasteur Institute, Lille, France; 5 Lille Nord de France University, Lille, France; 6 Division of Endocrinology and Metabolism, University of Pittsburgh School of Medicine, Pittsburgh, Pennsylvania, United States of America; 7 Department of Epidemiology and Biostatistics, Faculty of Medicine, Imperial College London, London, United Kingdom; 8 Centre de Recherche en Épidémiologie et Santé des Populations, INSERM U1018, Villejuif, France; 9 Université Paris-Sud 11, UMRS 1018, Villejuif, France; 10 Institute of Health Sciences, Biocenter Oulu, University of Oulu, Oulu, Finland; 11 Department of Genomics of Common Disease, School of Public Health, Imperial College London, Hammersmith Hospital, London, United Kingdom; University of Bremen, Germany

## Abstract

**Background:**

The *Nuclear Receptor 2F2* (*NR2F2*/*COUP-TFII*) heterozygous knockout mice display low basal insulinemia and enhanced insulin sensitivity. We previously established that insulin represses *NR2F2* gene expression in pancreatic β-cells. The *cis*-regulatory region of the *NR2F2* promoter is unknown and its influence on metabolism in humans is poorly understood. The present study aimed to identify the regulatory regions that control *NR2F2* gene transcription and to evaluate the effect of *NR2F2* promoter variation on glucose homeostasis in humans.

**Methodology/Principal Findings:**

Regulation of the *NR2F2* promoter was assessed using gene reporter assays, ChIP and gel shift experiments. The effects of variation at SNP rs3743462 in *NR2F2* on quantitative metabolic traits were studied in two European prospective cohorts. We identified a minimal promoter region that down-regulates NR2F2 expression by attenuating HNF4α activation in response to high glucose concentrations. Subjects of the French DESIR population, who carried the rs3743462 T-to-C polymorphism, located in the distal glucose-responsive promoter, displayed lower basal insulin levels and lower HOMA-IR index. The C-allele at rs3743462 was associated with increased NR2F2 binding and decreased *NR2F2* gene expression.

**Conclusions/Significance:**

The rs3743462 polymorphism affects glucose-responsive *NR2F2* promoter regulation and thereby may influence whole-body insulin sensitivity, suggesting a role of NR2F2 in the control of glucose homeostasis in humans.

## Introduction

NR2F2 is a nuclear receptor also known as the chicken ovalbumin upstream promoter transcription factor II (COUP-TFII). It exerts complex pleiotropic effects on glucose and lipid metabolism in various tissues and at different periods of life [1], [2], [3], [4], [5]. Li *et al.* observed that *NR2F2* heterozygous knockout mice displayed lower basal insulinemia and enhanced insulin sensitivity compared to wild type mice [4]. These observations suggest that variations in *NR2F2* gene expression might play an important metabolic function in rodents. Furthermore, we established that *NR2F2* gene expression is modulated by nutritional status in pancreatic β-cells [3], hepatocytes [3] and ventromedial hypothalamic neurons [6]. We also described, in pancreatic β-cells, a cross-regulation between NR2F2 and the transcription factor hepatocyte nuclear factor-4α (HNF4α), involved in the regulation of insulin secretion [7], [3]. These data further support a role for insulin and glucose in the control of NR2F2 and a pivotal role for this transcription factor in the control of glucose homeostasis *in vivo*.

The precise function of *NR2F2* in the control of glucose homeostasis in humans is poorly understood. We recently established the presence of *NR2F2* expression in human pancreatic β-cells [5]. To our knowledge, no specific mutations in *NR2F2* have been identified in relation to diabetes phenotypes and variations in the human *NR2F2* sequence have not previously been associated with changes in glucose metabolism in human populations. Such observations may provide further support for a role for *NR2F2* in the glucose-insulin metabolism in humans.

The present report aimed to identify and characterize some of the *cis*-regulatory regions of the *NR2F2* promoter that confer glucose responsiveness *in vitro* and *in vivo*. We used the glucose sensitive INS-1 832/13 pancreatic β-cell line to study the influence of high glucose concentrations on *NR2F2* responsiveness. We investigated the association between single nucleotide polymorphisms (SNPs) in the promoter region of *NR2F2* and quantitative traits related to glucose homeostasis in the French prospective DESIR cohort and followed-up the lead SNP, rs3743462, in the European birth cohorts NFBC-1966 and NFBC-1986. Finally, we used fusion gene and gel shift assays to characterize the consequences of the allelic change at rs3743462 on *NR2F2* promoter activity.

## Results

### High Glucose Concentrations in vitro Promote Repression of the Proximal Region of NR2F2 by Attenuation of the Activation Effect of HNF4α

**Figure 1:**
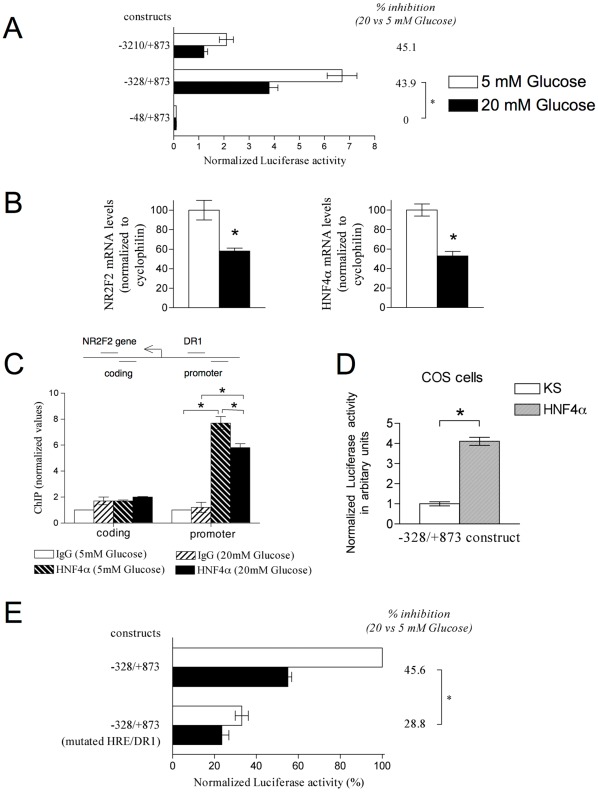
Glucose decreases the ability of HNF4α to activate the transcription of the *NR2F2* proximal promoter and to bind its chromatin target. (A) INS-1 832/13 cells were transiently co-transfected as described in [7] with a luciferase reporter gene driven by various lengths of the *NR2F2* promoter, designated by their 5′ and 3′ end positions relative to the defined *NR2F2* gene transcription initiation site [25] and a control vector expressing *Renilla* luciferase. Cells were cultured in the presence of either 5 mM (white bars) or 20 mM (black bars) glucose. Results were calculated from the ratio of luciferase/*Renilla* activity. Background expression was subtracted using the mean expression level of empty pGL3-basic. Means ± SEM of results obtained from at least three independent transfections performed in triplicate are shown. *Significant differences, P<0.05. (B) Comparison of mRNA levels of NR2F2 and HNF4α relative to those of cyclophilin determined by real-time RT-QPCR in INS-1 832/13 β-cells stimulated with 5 mM (white bars) or 20 mM glucose (black bars) for 24 h as described in [3] (C) ChIP from INS-1 832/13 cells cultured in the presence of either 5 mM or 20 mM glucose. Targets for QPCR amplifications were the proximal *NR2F2* promoter containing the DR1 DNA binding site or a downstream coding region (negative control). Amount of target chromatin precipitated by the HNF4α-specific antibody relative to that precipitated by control IgG (mean of three independent experiments ± SEM). *Significant difference, P<0.05. (D) COS cells were transiently co-transfected with the −328/+873 construct and 50 ng of empty KS vector (white bars), or HNF4α (grey bars) expression vectors. Means ± SEM of results from at least three separate transfections performed in triplicate are shown. *Significant difference, P<0.05. (E) INS-1 832/13 cells were transiently co-transfected with the wild-type or mutated −328/+873 constructs containing the DR1 elements. Cells were cultured in the presence of either 5 mM (white bars) or 20 mM (black bars) glucose. Means ± SEM of results from ten independent transfections performed in triplicate are shown. *Statistically significant differences in percentage of expression (where value for 5 mM glucose is 100% relative to −328/+873 construct) at P<0.05.

To map the *cis*-regulatory elements responsible for glucose responsiveness in *NR2F2*, we transiently co-transfected luciferase plasmid reporters in a INS-1 832/13 β-cell line with various deleted sections of the *NR2F2* regulatory region. This corresponds to a fragment of 4 kbp that encompasses the upstream *NR2F2* regulatory region that was shown to be sufficient to direct NR2F2 expression in pancreatic β-cells [8]. In comparison with a concentration of 5 mM, a glucose concentration of 20 mM provoked a 45% reduction of luciferase activity (*P<*0.05) in INS-1 832/13 β-cells co-transfected with the full length *NR2F2* promoter construct −3210/+873) (Fig. 1A). These measured impacts were consistent with previous observations on endogenous reduction of *NR2F2* mRNA abundance by high glucose concentrations (Fig. 1B and [3]). The deletion analysis further established that the fragment from −328 to +873 was the minimal region required to confer a significant inhibition by high glucose concentrations (Fig. 1A). In this proximal promoter, we previously showed that HNF4α binds the conserved direct repeat-1 (DR-1) hormone response element (HRE) [7]. We also showed that HNF4α is able to activate the endogenous *NR2F2* gene in INS-1 832/13 β-cells [7]. As shown in figure 1B, we observed the same reduction of HNF4α mRNA levels as observed for NR2F2 mRNA levels in the presence of high glucose levels in INS-1 cells suggesting that this transcription factor could be involved in the glucose responsiveness of *NR2F2.* As show in figure 1C, the antibodies for HNF4α immunoprecipitated with the promoter region of *NR2F2,* revealing the presence of endogenous HNF4α on this DNA binding site. A high glucose concentration (20 mM) in comparison to a concentration of 5 mM induced a significant reduction of immunoprecipitation with anti-HNF4α antibodies. HNF4α bound to the DR-1 DNA binding site in a glucose-dependent manner. In COS-7 cells, that lack endogenous expression of HNF4α, the co-transfection with an HNF4α expression vector and the −328/+873 luciferase reporter plasmid induced a 4-fold increase in luciferase activity (Fig. 1D). Moreover, when the DR-1 DNA binding site was mutated in the −328/+873 construct, we measured a 70% reduction in luciferase activity (Fig. 1E). This suggests that a nuclear receptor is a transactivator of the *NR2F2* promoter in β-cells cultured in 5 mM glucose, a condition that allows maximal expression of NR2F2. Furthermore the mutations at the DR-1 binding site led to a significantly weaker repression by 20 mM glucose (Fig. 1E). Altogether these results suggest that HNF4α is a transcription factor required for inhibition of *NR2F2* promoter activity by high concentrations of glucose. They also suggest a more complex pattern of regulation with additional factors involved in the inhibition of *NR2F2* transcription activity by high concentrations of glucose.

### Genetic Analysis at the Human NR2F2 Gene Locus

#### Discovery and genotyping in the DESIR cohort

**Figure 2:**
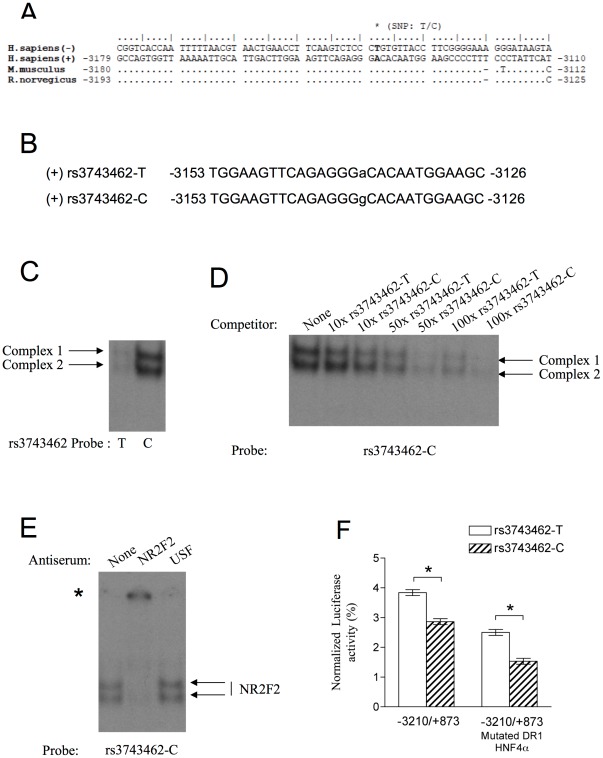
NR2F2 binds the variant rs3743462-C oligonucleotide with higher affinity than the rs3743462-T oligonucleotide and the C-allele is associated with a strong decrease of *NR2F2* gene expression relative to the T-allele. (A) Multiple alignments of the genomic region between nucleotides −3180 and −3110 of the *NR2F2* gene regulatory regions present in the −3210/+873 construct. Deletion is indicated by dashes and points indicate identities. The sequence of the human complementary strand is shown above other sequences. Genomic sequences can be retrieved from GenBank by their accession codes: *Homo sapiens* (NT_010274.17|:11836273-11840385), *Mus musculus* (NT_039428.7|Mm7_39468_37:c10507606-10503510; reverse/complementary strand), *Rattus norvegicus* (NW_047560.2|Rn1_WGA2082_4:c5641306-5636690; reverse/complementary strand). The position of the human SNP is indicated by an asteriskabove the sequences of each species: *H. sapiens*, −3,138; *M. musculus*, −3,139; *R. norvegicus*, −3,152 (where transcription start site is +1). (B) The sense strand sequences (+) of the oligonucleotides used in EMSA are shown. SNP base pairs are shown in lower case letters. (C) The labeled rs3743462-T and rs3743462-C oligonucleotides were incubated with INS-1 832/13 nuclear extracts, and protein binding was analyzed using EMSA. In the representative autoradiograph shown, only the retarded complexes are visible and not the free probe, which was in excess. (D) Comparison of the affinity of protein binding to the rs3743462-T and rs3743462-C variants. The labeled rs3743462-C oligonucleotide (Fig. 2B) was incubated with or without the indicated molar excess of unlabeled rs3743462-T or rs3743462-C oligonucleotide as competitors before addition of INS-1 832/13 nuclear extract. Protein binding was then analyzed using EMSA. In the representative autoradiograph shown, only the retarded complexes are visible and not the free probe, which was in excess. (E) INS-1 832/13 nuclear extracts were incubated with or without of the indicated anti-serum. The labeled oligonucleotide representing the −3153/−3126 *NR2F2* regulatory region and containing the rs3743462-C allele was added and protein binding was analyzed using EMSA. In the representative autoradiograph shown, only the retarded complexes are visible and not the free probe, which was in excess. (F) Functional analysis of the rs3743462 alleles in pancreatic β-cells. INS-1 832/13 cells were transiently co-transfected using lipofectamine solution containing either rs3743462 T-allele, C-allele, T-allele with DR-1 mutated site or C-allele with DR-1 mutated site within the context of the 3210/+873 sequences (1.5 µg) and expression vector encoding *Renilla* luciferase (0.1mg). Cells were then cultured in the presence of 5 mM glucose for 14 h. Results are calculated from the ratio of luciferase/*Renilla* activity. Means ± SEM of results obtained from at least three independent transfections performed in triplicate are shown. *Significant differences in expression at P<0.05.

In order to assess part of the functional basis of NR2F2 in relation to glucose homeostasis in humans, we have tested the hypothesis that variations in the allelic distribution of common SNPs at the NR2F2 locus impact glucose metabolism-related quantitative traits. Three common SNPs (rs3743462, rs1807198 and rs11045, with a minor allele frequency (MAF) >0.10), located in a 12.5-kbp genomic interval at the NR2F2 locus on human chromosome 15q26, were analyzed as previously described [9]. We first measured their association in a subset of 654 normoglycemic non-obese individuals selected from the prospective DESIR cohort [10]. Only one SNP, rs3743462, showed trends of association with lower fasting insulin plasma concentrations, lower indices of basal insulin secretion (HOMA-B) and insulin resistance (HOMA-IR) (P≤0.002). This gene variant is located in an upstream regulatory region of the NR2F2 gene, at −3,138 bp from the transcription start site (Fig. 2A) and the effect allele is characterized by the substitution of a thymidine by a cytosine. No strong linkage disequilibrium (LD) (r^2^<0.50 from the HapMap3 database) was seen between rs3743462 and 14 other common SNPs present over a 500 kbp region encompassing the NR2F2 locus.

The entire DESIR cohort, comprising 4,833 subjects of whom 3,877 were followed-up for 9 years [10], was genotyped for the *NR2F2*-rs3743462 variant. The frequency of the minor C-allele of rs3743462 was 14%. The association between *NR2F2*-rs3743462 and quantitative glucose homeostasis traits was evaluated in 3,341 non diabetic healthy individuals by using mixed regression models with data adjusted for age, sex and body mass index (BMI). The minor C-allele showed a significant negative association with fasting insulin levels (*P* = 0.005) and HOMA-IR index (*P* = 0.0026) (Table 1). When the regression was not adjusted for BMI, the association was of greater significance for both insulin and HOMA-IR (Table S1). No significant genotype correlation with the fasting glucose concentrations or the basal β-cell insulin secretion index (HOMA-B) was observed in the DESIR cohort (Table 1); only a weak trend of association was observed for HOMA-B when regression was not adjusted for BMI (Table S1). No association of *NR2F2*-rs3743462 was observed with weight, BMI or whole-body adiposity index (BAI) [11] in the DESIR cohort. We also observed an association between rs3743462 C-allele and height (*P* = 0.0026). No association with the prevalence or incidence of type 2 diabetes (T2D) was observed when we included incident diabetic cases during follow-up and individuals diabetic at entry into the study.

**Table 1:** Genotype correlation of *NR2F2* rs3743462 polymorphism on glucose homeostasis parameters and height in the French prospective DESIR cohort using up to four repeated measurements over the 9-year follow-up study.

	Number of observations	*P* value*	Effect size	Overall mean ± SD			
			β-coefficient (95% CI)*	TT		TC	CC
**Fasting plasma glucose (mmol/l)**							
additive	11,564	0.37	−0.008 (−0.026, 0.010)	5.30±0.52		5.28±0.53	5.26±0.46
dominant		0.51	−0.007 (−0.027, 0.013)				
recessive		0.28	−0.035 (−0.097, 0.028)				
**Fasting serum insulin (pmol/l)**							
additive	11,558	0.005	−2.498 (−4.199, −0.777)	50.68±31.95	48.66±34.18		48.21±29.86
dominant		0.008	−2.625 (−4.524, −0.688)				
recessive		0.10	−5.010 (−10.694, 1.035)				
**HOMA-IR**							
additive	11,530	0.003	−2.849 (−4.648, −1.005)	2.00±1.38	1.92±1.48		1.84±1.15
dominant		0.006	−2.878 (−4.896, −0.817)				
recessive		0.04	−6.695 (−12.672, −0.309)				
**HOMA-B**							
additive	11,520	0.10	−1.538 (−3.352, 0.3105)	102.71±69.40	102.53±96.80		97.23±63.15
dominant		0.16	−1.499 (−3.526, 0.582)				
recessive		0.19	−4.247 (−10.33, 2.255)				
**Height (cm)**							
additive	13,470	0.0026	0.316 (0.111, 0.522)	165.76±9.10	165.94±9.43		168.03 ± 9.57
dominant		0.006	0.323 (0.094, 0.553)				
recessive		0.052	0.723 (−0.007, 1.452)				

* The *P* values and β-coefficients are from the “mixed” regression model of each trait against genotype with age, gender and BMI as covariates (except for height adjusted for age and gender). The *P*-values indicated are nominal *P*-values.

The β-coefficient denotes the effect of rs3743462 minor C-allele and genotypes (depending on the genetic model tested) on the traits analyzed, i.e. the increase or decrease of the mean value for a specific trait.

### Follow-up Studies

We attempted to replicate these associations for fasting insulin, HOMA-IR and height in the population-based NFBC-1966 (n = 3,476 individuals with one measurement point at age 31 for all participants) (Table S2). Although we had a statistical power of 95% to detect the observed genetic effect size, no association was observed for the tested traits (*P*>0.05) in this population of Finnish ancestry. Similarly, no evidence of association was found in the NFBC-1986 and the MAGIC meta-analysis dataset [12]. We suspect these differences may be due to inter-cohort phenotypic and genetic heterogeneity. For instance, we observed a MAF of 14%, 22% and 17%, in the DESIR, NFBC-1966 (and NFBC-1986) and MAGIC studies, respectively.

### Characterization and Functional Analysis of the NR2F2-rs3743462 Variants in a Pancreatic β-cell line

The genetic variant rs3743462 is located in the distal promoter of *NR2F2*, a sequence highly conserved across species (Fig. 2A). We undertook a biochemical approach to assess whether allelic variation at the rs3743462 polymorphism could affect *NR2F2* promoter activity. We found this DNA region in a NR2F2/HRE motif. NR2F2 is considered to be the most promiscuous nuclear receptor that recognizes direct, inverted or palindromic repeats of the HRE motif “A/GGGTCA” with different interspaced distances [13], [14]. Electrophoresis mobility shift assay (EMSA) was used to investigate whether the protein NR2F2 binds to the *NR2F2* distal promoter region *in vitro*. ^32^P labeled double-stranded oligonucleotides, rs3743462-T and rs3743462-C probes (Fig. 2B) were incubated with INS-1 832/13 nuclear extracts. We identified two DNA-protein complexes (Fig. 2C) with higher affinity for the rs3743462-C oligonucleotide probe than the rs3743462-T (Fig. 2C). Competition with non-labeled DNA at various concentrations (Fig. 2D) further demonstrated that the rs3743462-C oligonucleotide competed more efficiently than the rs3743462-T oligonucleotide for the formation of DNA-protein complexes. In fact, quantification of the results from several experiments (data not shown) suggested that the protein binding capacity of the rs3743462-C oligonucleotide was approximately five-fold higher than for the rs3743462-T oligonucleotide. To further establish that NR2F2 was involved in the DNA-protein complexes, we then performed EMSA with nuclear extracts of INS-1 832/13 cells, preincubated with antisera specific to NR2F2 or the upstream stimulatory factor (USF) used as a control [15]. As shown in Fig. 2E, incubation with antibodies specific to NR2F2 resulted in a supershift in the migration of both complexes. A pre-incubation with anti-USF had no effect (Fig. 2E). This result strongly supported the fact that the DNA-protein complexes are characterized by binding of NR2F2. Finally, together with the previous observations showing a higher capacity of the variant rs3743462-C oligonucleotide to create these complexes in comparison to the rs3743462-T oligonucleotide *in vitro* (Fig. 2C and Fig. 2D), this suggests that rs3743462-C has a greater affinity to bind with NR2F2.

We next investigated the functional impact of altered NR2F2 binding on the *NR2F2* promoter activity in β-cells cultured in 5 mM glucose, the condition that allows maximal expression of *NR2F2*. We generated luciferase reporter plasmids controlled by either the rs3743462-C or -T allele in the −3210/+873 *NR2F2* promoter region. Figure 2F shows that the presence of the rs3743462-C allele was associated with a 26% reduction in the activity of the promoter in comparison to the rs3743462-T allele (*P*<0.05). Finally, we mutated the DR-1/HNF4α binding site in the construct with the rs3743462-C or -T alleles in the −3210/+873 *NR2F2* promoter region (Fig. 2F). The addition of the DR1 mutated DNA binding site to the rs3743462-C allele led to a lower transcriptional activity. Nonetheless, the amplitude of changes in luciferase activity between the two allele constructs was not affected indicating that the rs3743462-C allele that binds NR2F2 does not require a functional DR1-HNF4α site in order to repress transcription.

## Discussion

Several recent studies on rodent models suggest that NR2F2 is involved in glucose homeostasis and in energy metabolism ([1], [3], [4], [5] and L. Sabra-Makke *et al.* in preparation). Interestingly, *NR2F2* is also regulated by nutrients and hormones [3], [6]. For instance, its expression is repressed directly in response to exogenous insulin and indirectly in response to high glucose concentrations through enhanced insulin secretion in pancreatic β-cells [3].

In the present study, we showed that the proximal promoter of *NR2F2* is responsive to changes in glucose concentrations. In INS-1 pancreatic β-cells, high glucose concentrations decreased both the HNF4α-dependent ChIP signal on the DR-1 DNA binding site and a DR-1-driven luciferase reporter gene transactivation. These findings were further supported by the co-repression of *HNF4α* and *NR2F2* genes in the presence of 20 mM glucose concentrations in INS-1 β-cells as previously described for HNF4α expression in hepatocytes [16]. Altogether our data suggest that the activation of *NR2F2* gene expression in the fasted state, when insulin and glucose concentrations are at their lowest, results in part from the stimulation of HNF4α. Furthermore, we showed that allelic variation at rs3743462 in a conserved *cis*-regulatory region of the distal *NR2F2* promoter is associated with fasting insulin concentrations, the HOMA-IR index and adult height in non diabetic individuals in the DESIR study. These three phenotypic traits have previously been associated with metabolic health in various populations [17]. The DESIR prospective cohort is a comprehensively phenotyped general population of middle-aged individuals, in which we previously analyzed several genetic variants with confirmed significant effects either on T2D risk or on glucose and lipid homeostasis quantitative traits [18], [19], [20], [21]. By assessing a genotype effect on continuous variables measured over the 9-years of follow-up in the study, we are quite confident of the validity of the associations between rs3743462 and the metabolic variables tested in this large general population. However, our follow-up study of these genetic associations in other European populations did not replicate these observations. The latest meta-analyses of fasting insulin and HOMA-IR from the MAGIC consortium dataset did not show evidence of associations for rs3743462 [12]. Furthermore, the GIANT meta-analysis of adult BMI did not reveal any association at genome-wide level significance between rs3743462 and adult BMI [22]. The allelic change from T to C at the *NR2F2*-rs3743462 polymorphism probably has modest effects on glucose homeostasis *in vivo*. Such effects are very likely dependent on complex gene-environment interactions that have yet to be disentangled. Nevertheless, observation from the DESIR study, that variation within the distal promoter region of *NR2F2* is associated with whole-body insulin sensitivity, is partly consistent with the phenotype of *NR2F2* heterozygous knockout mice [4] which show improved glucose homeostasis and increased energy expenditure. These mutant mice displayed normal fasted plasma glucose, lower fasted insulin concentrations and improved insulin sensitivity.

Interestingly, we showed in a pancreatic β-cell line that the presence of the rs3743462-C allele enhances *NR2F2* binding to its distal promoter. Our *NR2F2* fusion gene assay with the C-allele of the SNP resulted in a repression of gene activity compared to the T allele suggesting that the binding of NR2F2 to its distal promoter participates in decreasing *NR2F2* expression. These observations are consistent with previous experiments where over-expression of NR2F2 decreased endogenous NR2F2 mRNA abundance in pancreatic β-cells [7], suggesting a possible negative feedback of NR2F2 on its own gene transcription. It would be interesting to test NR2F2 binding *in vivo* but since it is the mutation that promotes increased NR2F2 binding it is not easy to realize these experiments.

We have shed light on the importance of both HNF4α and NR2F2 itself in the control of its expression and suggested an HNF4α-dependent site that contributes to regulate *NR2F2* transcription in response to glucose concentrations changes. Our data are suggestive of a greater affinity of NR2F2 to control its own promoter in the presence of the rs3743462-C allele that is associated with whole-body insulin sensitivity in a general population of non diabetic individuals. The role of NR2F2 regulation in the aetiology of complex metabolic diseases, such as the metabolic syndrome and T2D has yet to be fully understood. In conclusion, our data suggest that NR2F2 might be a key factor in novel strategies aiming to prevent or treat some of the metabolic defects related to insulin resistance in humans.

## Methods

### Ethical Statements

All subjects included in the DESIR and NFBC 1966 studies gave written consent, and the protocol was approved by the ethics committee of Bicêtre Hospital, Kremlin-Bicêtre, France for DESIR and the University Hospital of Oulu in accordance with the declaration of Helsinki for NFBC 1966.

### Study Cohorts

Participants in DESIR (Data from an Epidemiological Study on the Insulin Resistance syndrome), a prospective study of a middle-aged French cohort fully described elsewhere [10], were clinically and biologically evaluated at inclusion and at 3-year, 6-year and 9-year follow-up visits [18]. Of the 4,833 individuals of European ancestry included, 3,877 were examined at each 3-year follow-up visit over the entire 9-year study. This number includes 3,570 non diabetic individuals who did not receive treatment with hypoglycemic drugs over the period of the study. All subjects included in the study gave their informed consent, and the protocol was approved by the ethics committee of Bicêtre Hospital, Kremlin-Bicêtre, France.

The population-based cohort Northern Finland Birth Cohort (NFBC)-1966 is a genetically homogeneous birth cohort of white Caucasians born in the two northernmost provinces of Finland (Oulu and Lapland). It consists of an unselected population of white Caucasian mothers and their offspring with expected dates of delivery from between end of 1965 and early in 1967 [23]. Extensive growth measurements and clinical examinations of the offspring were conducted from birth until age 31. At age 31, fasted blood samples were withdrawn from the offspring for metabolic profiling and genotyping. The present study included the data of singleton term-born offspring. All individuals included in the present study (n = 3,476; male-to-female ratio = 1) were normoglycemic (fasted glycemia ≤5.6 mmol/l) with normal BMI (≤25 kg/m^2^ for all participants). All participants and their parents gave written informed consent. The study protocol has been approved by the ethics committee of the Faculty of Medicine of the University of Oulu and the Finland Ministry for Social and Health Affairs.

### SNP Genotyping

Genotyping of rs3743462 in the DESIR cohort was performed using TaqMan Technology (assay n°C-395241-10, Applied Biosystems, Foster City, CA, USA). A genotyping success rate of 98.8% was achieved in samples from the whole cohort. Duplicate samples were assayed with a concordance rate of 100%. The genotype distribution of rs3743462 was in Hardy-Weinberg equilibrium (*p*>0.20). The genotype data for rs3743462 in the NFBC-1966 cohort were available from a previously published genome-wide study [24].

### Genetic and Statistical Analyses

In the DESIR cohort, the correlation between rs3743462 genotypes and quantitative parameters was assessed using data from untreated individuals both at inclusion and 3, 6 and 9 years later [18]. The normoglycemic status was defined as a fasting plasma glucose (FPG) of <6.1 mmol/l in the absence of hypoglycemic treatment (according to 1997 American Diabetes Association criteria). In the NFBC-1966 cohort, the effect of rs3743462 was tested on the measurements at age 31 years in all included participants.

The quantitative trait data were log-transformed before analysis (when required) and adjusted for age and sex, and BMI when specified. We used linear regression models for analyses of one measurement point and mixed regression models for analyses of follow-up data [18] with adjustment for co-variables (age and/or sex and BMI). All analyses were performed using R (Rproject.org).

### Plasmids and Site-directed Mutagenesis

In the following description, in construct names and throughout the paper, when referring to *NR2F2* gene structure, numbers designate the position of nucleotides relative to the known transcription initiation site at +1 [25]. The mouse and human regulatory regions studied are nearly identical (see Figure 2A). The reporter plasmids −328/+873 and −48/+873 (corresponding to −328/luc and −48/luc respectively) and −328 mutated DR-1 site/+873 (−328M/luc) have been previously described [7]. The reporter plasmids −3210/+873 (T-allele at rs3743462) and −3210/+873 (C-allele at rs3743462) were obtained by cloning a synthesized 493-bp mouse COUP-TFII fragment between the KpnI (−3210) and PvuII (−2717) sites (GENEART AG, Regensburg) into the −3000/+873 plasmid (−3000/luc), described in [7], digested with KpnI and PvuII. Site-directed mutagenesis of the reporter plasmids −3210/+873 (T- and C-alleles at rs3743462) in the DR-1 site was performed by GeneCust, Europe). All new constructs were completely sequenced. The *Renilla* luciferase plasmid, the pcDNA plasmid encoding HNF4α has been previously described [7].

### Cell Culture, Transfection and Reporter Gene Assay

Transfection and reporter gene assays were done as previously described [7]. Rat pancreatic INS-1 832/13 cells generously provided by C. Newgard (×10^6^/well) [7] or COS-7 fibroblast cells (Invitrogen) were transiently transfected, using lipofectamine 2000 reagent (Invitrogen), with the luciferase reporter gene downstream of different portions of the mouse *NR2F2* promoter (0.45 µg of DNA) and a control vector expressing *Renilla* luciferase (0.1 µg of DNA). Transfected cells were cultured in 5 mM or 20 mM glucose as described [3] and harvested 14 h after transfection. Where necessary, transcriptional efficiency was first determined by transfecting different amounts of the reporter constructs; 1.5 µg of DNA and 0.1 µg of control vector expressing *Renilla* were optimal for obtaining significantly different activities between constructs. Cell extracts were assayed for reporter enzyme activities using the Dual-Luciferase Reporter Assay System kit (Promega). Results were calculated as the ratio of luciferase/*Renilla*. Background expression, defined as the mean relative expression level of the empty pGL3-basic construct, was subtracted from all other values. The means ± standard errors of the mean (SEM) represent data from five to ten independent transfections performed in triplicate.

### Nuclear Extract Preparation and EMSA

Nuclear extracts from 832/13 INS-1 cells were prepared as described [7]. Electrophoresis mobility shift assays (EMSA), probe labeling, binding reactions, antibody supershift analyses were performed as reported previously [7]. Protein binding was quantified by using Typhoon (GE Healthcare) to count the proportion of ^32^P present in retarded complexes.

### Chromatin Immunoprecipitation

Chromatin immunoprecipitation (ChIP) assays were performed as previously described [26] with a slight modification of the Upstate Biotechnology (Millipore) ChIP Assay Kit protocol (catalog no. 17–295). Cells were treated for 16 h in 5 mM glucose prior to a 6-h treatment with 20 mM glucose. Extracted chromatin was sonicated using a Diagenode Bioruptor (Liege, Belgium) for 15 min. Antibodies from Santa Cruz Biotechnology used in ChIP experiments were HNF4α (H-171) (catalog no. sc-8987) and IgG (catalog no. sc-2027). Sequences of the primers used are: 5′-TGAACTTTGACACGACTGCTG-3′ and 5′-GCTAGGACCGGGCTGTTC-3′ for the COUP-TFII promoter; and 5′-CAGCAGCAGCACATCGAG-3′ and 5′-GGCAGTACTGGCACTGGTTG-3′ for the COUP-TFII coding region.

### Statistical Analysis

Quantitative results are expressed as means ± SEM. Statistical analyses were carried out using the Mann-Whitney test, a nonparametric statistical test appropriate when the sample number is less than 10. Null hypotheses were rejected at P values of >0.05.

## Supporting Information

Table S1 **Genotype correlation of** ***NR2F2*** **rs3743462 polymorphism on glucose homeostasis parameters and BMI in the French prospective DESIR cohort (repeated measures without adjustment for BMI).**
(DOC)

Table S2 **Genotype correlation of** ***NR2F2*** **rs3743462 polymorphism on glucose homeostasis parameters and height in the NFBC-1966 cohort.**
(DOC)
