# Supplementary material for: Glucose-Dependent Regulation of NR2F2 Promoter and Influence of SNP-rs3743462 on Whole Body Insulin Sensitivity
Source: PLoS One. 2012 May 14;7(5):e35810. doi: 10.1371/journal.pone.0035810 (PMC3351448; doi:10.1371/journal.pone.0035810)
Supplement: Table S1 — Genotype correlation of NR2F2 rs3743462 polymorphism on glucose homeostasis parameters and BMI in the French prospective DESIR cohort (repeated measures without adjustment for BMI). (DOC) [file pone.0035810.s001.doc]

**Table S1. Genotype correlation of *NR2F2* rs3743462 polymorphism on glucose homeostasis parameters and BMI in the French prospective DESIR cohort (repeated measures without adjustment for BMI).**

|  | Number of observations | *P* value* | Effect size | Overall mean ± SD | | | | |
| --- | --- | --- | --- | --- | --- | --- | --- | --- |
|  |  |  | β-coefficient (95% CI)* | **TT** | **TC** | | | **CC** |
|  |  |  |  |  | | | | |
| **Fasting plasma glucose (mmol/l)** |  |  |  |  | | | | |
| additive | 11,570 | 0.15 | -0.013 (-0.032, 0.005) | 5.30 ± 0.52 | 5.28 ± 0.53 | | | 5.26 ± 0.46 |
| dominant |  | 0.19 | -0.014 (-0.034, 0.007) |  | | | | |
| recessive |  | 0.35 | -0.031 (-0.095, 0.033) |  | | | | |
| **Fasting serum insulin (pmol/l)** |  |  |  |  | | | | |
| additive | 11,564 | 0.0002 | -3.709 (-5.626, -1.754) | 50.68 ± 31.95 | 48.66 ± 34.18 | | | 48.21 ± 29.86 |
| dominant |  | 0.0002 | -4.228 (-6.349, -2.049) |  | | | | |
| recessive |  | 0.237 | -4.161 (-10.685, -2.829) |  | | | | |
| **HOMA-IR** |  |  |  |  | | | | |
| additive | 11,536 | 0.00012 | -4.132 (-6.171, -2.058) | 2.00 ± 1.38 | 1.92 ± 1.48 | | | 1.84 ± 1.15 |
| dominant |  | 0.00014 | -4.563 (-6.826, -2.235) |  | | | | |
| recessive |  | 0.105 | -6.077 (-12.934, 1.329) |  | | | | |
| **HOMA-B** |  |  |  |  | | | | |
| additive | 11,526 | 0.014 | -2.479 (-4.400, -0.509) | 102.71 ± 69.40 | 102.53 ± 96.80 | | | 97.23 ± 63.15 |
| dominant |  | 0.016 | -2.712 (-4.867,-0.509) |  | | | | |
| recessive |  | 0.28 | -3.806 (-10.345, 3.210) |  | | | | |
| **BMI (kg/m^2^)** |  |  |  |  | | | | |
| additive | 13,470 | 0.17 | -0.329 (-0.817, 0.150) | 25.09 ± 3.75 | | 24.96 ± 3.77 | 25.09 ± 3.83 | |
| dominant |  | 0.09 | -0.459 (-0.995, 0.070) |  | | | | |
| recessive |  | 0.59 | 0.471 (-1.232, 2.214) |  | | | | |
|  |  |  |  |  | | | | |

*The *P* values and β-coefficients are from the “mixed” regression model of each trait against genotype with age and gender as covariates. The *P*-values indicated are nominal *P*­-values.

The β-coefficient denotes the effect of rs3743462 minor C-allele and genotypes (depending on the genetic model tested) on the traits analyzed, i.e. the increase or decrease of the mean value for a specific trait.
