# Supplementary material for: Glucose-Dependent Regulation of NR2F2 Promoter and Influence of SNP-rs3743462 on Whole Body Insulin Sensitivity
Source: PLoS One. 2012 May 14;7(5):e35810. doi: 10.1371/journal.pone.0035810 (PMC3351448; doi:10.1371/journal.pone.0035810)
Supplement: Table S2 — Genotype correlation of NR2F2 rs3743462 polymorphism on glucose homeostasis parameters and height in the NFBC-1966 cohort. (DOC) [file pone.0035810.s002.doc]

**Table S2.** **Genotype correlation of *NR2F2* rs3743462 polymorphism on glucose homeostasis parameters and height in the NFBC-1966 cohort**

|  | N | *P* value* | Effect size | Overall mean ± SE | | |
| --- | --- | --- | --- | --- | --- | --- |
|  | subjects |  | β-coefficient (SE)* | **TT** | **TC** | **CC** |
|  |  |  |  |  | | |
| **Fasting plasma glucose (mmol/l)** |  |  |  |  |  |  |
| additive | 3,476 | 0.36 | -0.009 (0.0093) | 4.904 ± 0.024 | 4.904 ± 0.010 | 4.910 ± 0.007 |
|  |  | 0.61^§^ |  |  |  |  |
|  |  |  |  |  |  |  |
| **Fasting serum insulin (mU/l)** |  |  |  |  |  |  |
| additive | 3,476 | 0.58 | -0.0378 (0.0724) | 7.345 ± 0.167 | 7.729 ± 0.078 | 7.633 ± 0.059 |
|  |  | 0.85^§^ |  |  |  |  |
|  |  |  |  |  |  |  |
| **HOMA-IR** |  |  |  |  |  |  |
| additive | 3,476 | 0.56 | -0.005 (0.0092) | 0.949 ± 0.021 | 0.998 ± 0.010 | 0.986 ± 0.007 |
|  |  | 0.84^§^ |  |  |  |  |
|  |  |  |  |  |  |  |
| **HOMA-B** |  |  |  |  |  |  |
| additive | 3,476 | 0.89 | 0.098 (0.656) | 95.58 ± 1.537 | 98.93 ± 0.711 | 97.73 ± 0.510 |
|  |  | 0.81^§^ |  |  |  |  |
|  |  |  |  |  |  |  |
| **Height (cm)** |  |  | -0.174 (0.181) | 171.9 ± 0.726 | 170.7 ± 0.262 | 171.0 ±0.201 |
| additive | 3,476 | 0.35 |  |  |  |  |
|  |  | 0.89^§^ |  |  |  |  |
|  |  |  |  |  | | |

The subjects analyzed are normoglycemic (fasting glucose levels below 5.6 mmol/l) non obese individuals from the NFBC-1966 cohort (all traits were measured at age 31 in all participants).

*The *P* values and β-coefficients are from a linear regression model of each trait against genotype with gender and BMI as covariates; ^§^*P* value with data non adjusted for gender and BMI. The *P*-values indicated are nominal *P*­-values.

The β-coefficient denotes the effect of rs3743462 minor C-allele on the traits analyzed, i.e. the increase or decrease of the mean value for a specific trait.
